# Serum 25-OH Vitamin D in relation to Bone Mineral Density and Bone Turnover

**DOI:** 10.1155/2014/487463

**Published:** 2014-07-07

**Authors:** Nicola Napoli, Rocky Strollo, Delia Sprini, Ernesto Maddaloni, Giovam Battista Rini, Enrico Carmina

**Affiliations:** ^1^Endocrinology and Diabetes, Universitá Campus Bio-Medico di Roma, Via Alvaro del Portillo 21, 00128 Rome, Italy; ^2^Dipartimento di Medicina Interna e Specialistica (DIMIS), Università di Palermo, Via del Vespro 129, 90127 Palermo, Italy; ^3^Endocrine Unit, Department of Medical and Biological Sciences, Universitá di Palermo, Via delle Croci 47, 90139 Palermo, Italy

## Abstract

It is unclear which vitamin D status is optimal for bone health. In this study, we aimed to assess cutoffs of 25-hydroxyvitamin D (25OHD) derived by the literature (20, 25, or 30 ng/mL) in relation to bone turnover and bone mineral density (BMD). Serum 25OHD, PTH, osteocalcin, bone alkaline phosphatase, and C-telopeptide were measured in 274 consecutive postmenopausal women. BMD of the lumbar spine (L1–L4) and of femoral neck were also evaluated. 50 patients had normal BMD, while 124 had osteopenia and 100 had osteoporosis. 37.6%, 56.2%, and 70.8% subjects had serum 25OHD lower than 20, 25, or 30 ng/mL, respectively. No differences in bone turnover markers were found when comparing patients with low 25OHD defined according to the different cutoffs. However, a cutoff of 25 ng/mL appeared to differentiate better than a cutoff of 30 ng/mL in those subjects with reduced femoral neck BMD. The PTH plateau occurred at 25OHD levels of 26–30 ng/mL. In conclusion, vitamin D deficiency is common in Sicilian postmenopausal women and it may be associated with low BMD and increased bone turnover markers. Further studies are needed to better define the right cutoff for normal vitamin D levels in postmenopausal women.

## 1. Introduction

Vitamin D deficiency causes defects of bone mineralization and low vitamin D status has been detected in patients with hip fractures [1–3]. While in the past it was thought that vitamin D deficiency affects mostly northern countries [4, 5] and where there is a restricted exposure to sunlight or in elderly patients [6, 7], other studies have shown that vitamin D deficiency may be common also in subtropical countries [3, 8, 9] or southern Europe [10] including Italy [11]. In a large clinical trial on raloxifene, it was found that a vitamin D deficiency is common in southern Europe (8.3% of the patients) [10]. In the same study, 24.3% of the postmenopausal women had low-normal vitamin D status, in a range that could be considered partial vitamin D deficiency [10]. Several studies have shown a negative correlation between BMI and vitamin D at any ages and in different clinical conditions. Therefore, the increasing prevalence of obesity and metabolic syndrome, which are associated with decreased bioavailability of dietary and cutaneously synthesized vitamin D, is an additional factor contributing to the widespread of vitamin D deficiency [12]. It should be noted that vitamin D deficiency is associated with muscle impairment and it is one of the contributing factors of a clinical condition known as “sarcobesity.”

However, there is no consensus on which levels of serum 25-hydroxyvitamin D (25OHD) should be considered abnormal [13–15]. In this study, we aimed to assess whether different cutoffs of 25OHD-deficiency are associated with altered bone turnover or bone mineral density (BMD) in a homogeneous population of postmenopausal women living in Sicily. Sicily is the most southern part of Italy; it is surrounded by the Mediterranean Sea and characterized by sun exposure for 2/3 of the year.

## 2. Experimental Subjects and Methods

### 2.1. Study Subjects

We enrolled 274 consecutive postmenopausal women, aged 48–65 years (mean age 57.7 ± 0.4), who, from December to May, were referred to our outpatient clinic at University of Palermo, for osteoporosis assessment. Patients with hyperparathyroidism, Paget's bone disease, or secondary osteoporosis were not included in the study. We also excluded patients who were previously treated for osteoporosis or were taking calcium or vitamin D. In all postmenopausal women a fasting blood sample was taken in the morning for measurement of 25OHD, PTH, osteocalcin (OC), bone alkaline phosphatase (BAP), and C-telopeptides (CTX). All measurements were performed during winter-spring season (from December to May). Informed consent was obtained before enrollment and the protocol was approved by ethical committee of University of Palermo.

### 2.2. Bone Mineral Density Evaluation

BMD of the lumbar spine (L1–L4) and of femoral neck (F) was determined using dual X-ray absorptiometry (DEXA, Lunar DPX-Plus).

### 2.3. Biochemistry

25OHD was measured using enzyme-linked immunosorbent assay (ELISA) using materials provided by Immunodiagnostic Systems (Boldon, United Kingdom). Intact PTH was measured by ELISA using materials provided by Biosource, Belgium. OC and CTX were measured by ELISA using materials provided by Biotech A/S (Herlev, Denmark). BAP was evaluated by ELISA using materials provided by Beckmann-Coulter (CA, USA). In all assays, the intra-assay coefficient of variation was 6% or less, and the interassay coefficient of variation was 15% or less.

### 2.4. Statistical Analysis

Analysis of variance and the Mann-Whitney *U* test were used for group comparisons. *P* less than 0.05 were considered statistically significant. Results were expressed as mean ± SD.

## 3. Results

Clinical and biochemical features of the studied population are shown in Table 1. 50 patients had normal BMD, while 124 patients had osteopenia (*T*-score between −1 and −2.5 SD) and 100 patients had osteoporosis (*T*-score ≤ −2.5 SD). In our population, mean 25OHD was 26.04 ± 10.14 ng/mL and 63 study subjects (23%) had serum 25OHD lower than 16 ng/mL (1 SD below 25OHD mean values). BMD and bone turnover were compared between subgroups delineated by different serum 25OHD levels. These cutoffs were based on literature data and were set to 20 ng/mL [13], 25 ng/mL [16], and 30 ng/mL [14].

Using a cutoff of 20 ng/mL, 103 study subjects (37.6%) had vitamin D deficiency. The prevalence of vitamin D deficiency increased to 56.2% (154 patients) using the 25 ng/mL cutoff and to 70.8% (174 patients) using the 30 ng/mL cutoff.

As shown in Table 2, study subjects with low serum 25OHD had higher serum PTH, BAP, and CTX, independently, on used cutoffs. In general, subjects with low serum 25OHD showed lower* T*-score independently of used cutoff, but this difference was lost on femoral neck when the cutoff at 30 ng/mL was used (−1.4 ± 1.0 versus −1.5 ± 1.0 SD). Therefore, a 25OHD level higher than 25 ng/mL appeared to differentiate better than a cutoff of 30 ng/mL in those subjects with reduced femoral neck BMD.

**Figure 1:**
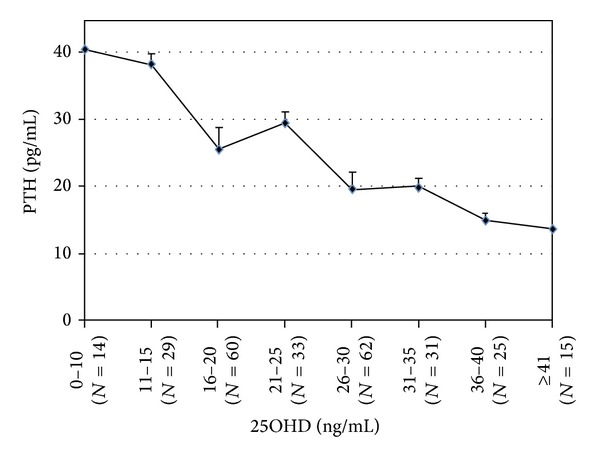
Mean (±SE) PTH by 25OHD subgroups. The graph shows subject PTH serum levels according to serum 25OHD subgroups defined by specific cutoffs. No clear inflection point was evident for the 25OHD cutoffs studies. However, there was a 34% increase in PTH levels (19.5 ± 1.53 versus 29.5 ± 3.3 pg/mL; *P* = 0.002) when comparing the two subgroups delineated by the 25OHD cutoff of 25 ng/mL. PTH levels did not change significantly differently when comparing the subgroups delineated by the 25OHD levels of 20 ng/mL (29.5 ± 3.3 versus 25.67 ± 1.51 pg/mL; *P* = 0.228), 30 ng/mL (19.5 ± 1.53 versus 20.06 ± 2.61 pg/mL; *P* = 0.853), 35 ng/mL (14.96 ± 1.28 versus 20.06 ± 2.61 pg/mL; *P* = 0.110), or 40 ng/mL (13.8 ± 1.16 versus 14.96 ± 1.28 pg/mL; *P* = 0.543), respectively.

No significant differences in mean values of OC, BAP, and CTX were found when subjects considered as 25OHD-deficient, according to the different cutoffs, were compared. However, by plotting serum 25OHD to PTH levels, there was a significant 34% increase in PTH levels (19.5 ± 1.53 versus 29.5 ± 3.3; *P* = 0.002) when comparing the two subgroups delineated by the 25OHD cutoff of 25 ng/mL (Figure 1); on the other hand, PTH levels did not change significantly when comparing the subgroups delineated by the 25OHD levels of 20 ng/mL (29.5 ± 3.3 versus 25.67 ± 1.51 pg/mL; *P* = 0.228), 30 ng/mL (19.5 ± 1.53 versus 20.06 ± 2.61 pg/mL; *P* = 0.853), 35 ng/mL (14.96 ± 1.28 versus 20.06 ± 2.61 pg/mL; *P* = 0.110), or 40 ng/mL (13.8 ± 1.16 versus 14.96 ± 1.28 pg/mL; *P* = 0.543), respectively.

## 4. Discussion

There is no consensus on what levels of serum 25OHD should be considered abnormal [13–15], in part because vitamin D needs vary among different ethnic groups and geographical areas and also because there is limited data on which levels of 25OHD are associated with subtle abnormalities of bone metabolism, turnover, and neuromuscular function. The Institute of Medicine has set the optimal 25OHD level at 20 ng/mL (corresponding to 2 SD above the median needs) as it was suggested to meet the requirement of at least 97.5% of population in North America [13]. However, there is still some controversy about optimal levels [14] and the International Osteoporosis Foundation recommends a desirable 25OHD serum level of 30 ng/mL [15].

This issue is particularly difficult when studying populations with possible vitamin D deficiency. It is still an open question if the optimal cutoff should be obtained in the same population or should be derived from literature and obtained in populations with different genetic and environmental influences. We tried to answer this question studying a Sicilian population of postmenopausal women. While this cannot be considered an epidemiological study, it is representative of the women who come to an osteoporotic clinic for the assessment of their bone mass.

All cutoffs divided the population in two groups different for* T*-score, bone turnover, and PTH levels. For any analyzed cutoffs, BMD was generally lower in the vitamin D deficient groups with consequent significant increase in PTH. Both markers of bone resorption and formation resulted higher in the vitamin D deficient groups, indicating an increased bone turnover. These data confirm that, despite the chosen cutoff, lower vitamin D levels may always negatively affect bone health. Subjects with low serum 25OHD showed lower* T*-score independently of used cutoff, but this difference was lost on femoral neck when the cutoff at 30 ng/mL was used. Moreover, by plotting serum 25OHD to PTH levels, a significant change in PTH levels was evident when comparing the two subgroups delineated by the 25OHD cutoff of 25 ng/mL but not for higher or lower 25OHD cutoffs, suggesting that a plateau occurred at 26–30 ng/mL. This suggests that a status of vitamin D deficiency exists in women having vitamin D lower than 20 or 25 ng/mL while the level of 30 ng/mL may be too high. In fact, using this cutoff 2/3 of studied women could be considered as having a vitamin D deficiency. Our data are consistent with the finding of the National Health and Nutrition Survey (NHANES) III where the risk of hip fracture was significantly reduced among participants with 25OHD levels greater than 25 ng/mL compared with those who had lower concentrations, and the association resulted to be independent of bone density [16]. However, our data should be read with caution because the number of people included in this study, and particularly those with 25OHD higher than 30 ng/mL, is relatively small. Furthermore, although we found a significant change in PTH levels for 25OHD at 26–30 ng/mL, an inflection point was not clearly evident. In another study on a larger sample, Holick et al. found that an inflection point for PTH levels is evident for 25OHD less than 29.8 ng/mL [17]. On the other hand, our data corroborate recent findings showing that vitamin D deficiency is common in postmenopausal women living in Mediterranean countries [10] including Italy [18], despite the general belief that this condition is common only in elderly patients [6, 7] or in countries where exposure to sunlight is low and limited to short periods of the year [4, 5]. While the reasons for this are not clear, possibly a poorer intake or darker skin in Mediterranean population, our study supports the idea that vitamin D status should be assessed in all postmenopausal women. A number of cross-sectional studies have found a positive association between 25OHD and BMD in postmenopausal women [5, 19–23], and the last NHANES in US [13] as well as a recent Italian study [18] showed that this relationship can be evident even in women before the onset of menopause. Interestingly, in the Italian study 25OHD levels were significantly lower in women from south sites compared with northern sites, despite a significantly higher sun exposure [18]. Moreover, impaired vitamin D status has been generally associated with an increased risk of fractures. A nested case control study from the Women's Health Initiative showed a near doubling of the odds ratio of risk for hip fracture in subjects with 25OHD lower than 20 ng/mL [24].

In conclusion, it is challenging to determine a precise cutoff for vitamin D deficiency in postmenopausal women, but, according to our study, a level of 25 ng/mL might be optimal. However, vitamin D deficiency causes bone loss and increased bone turnover and, therefore, vitamin D status should be assessed and corrected in populations at risk.

## Figures and Tables

**Table 1:** Clinical and biochemical features and *T*-scores of studied population. Data are mean ± standard error.

Age (years)	57.7 ± 0.8
BMI (Kg/m^2^)	26.6 ± 0.4
25OHD (ng/mL)	26.04 ± 1.9
L1–L4 (SD)	−0.5 ± 0.01
Femoral neck (SD)	−0.4 ± 0.01
PTH (pg/mL)	27.6 ± 1.1
OC (ng/mL)	14.8 ± 0.9
BAP (*μ*g/L)	18 ± 0.9
CTX (pmol/L)	2893 ± 154

**Table 2:** Features, *T*-scores, and biochemical markers of subjects subdivided three times into two groups on the basis of the different 25OHD cutoff values (20, 25, and 30 ng/mL).

	Cutoff at 20 ng/mL		Cutoff at 25 ng/mL		Cutoff at 30 ng/mL	
	25OHD >20 ng/mL (*n* = 171)	25OHD <20 ng/mL (*n* = 103)	25OHD >25 ng/mL (*n* = 120)	25OHD <25 ng/mL (*n* = 154)	25OHD >30 ng/mL (*n* = 80)	25OHD <30 ng/mL (*n* = 194)
Age (years)	57.6 ± 6.4	57.1 ± 6.0	56.6 ± 5.5	57.8 ± 6.2	56.5 ± 6.0	57.7 ± 6.3
BMI (Kg/m^2^)	26.6 ± 4.4	27.0 ± 3.5	25.9 ± 3.7	27.4 ± 4.1	25.9 ± 3.6	27.2 ± 4.2
Lumbar (L1–L4) *T*-score	−1.9 ± 1.3∗∗	−2.2 ± 1.3	−1.7 ± 1.3∗∗	−2.2 ± 1.6	−1.7 ± 1.3∗∗	−2.1 ± 1.2
Femoral neck *T*-score	−1.2 ± 1.0∗∗	−1.8 ± 1.0	−1.3 ± 1.2∗∗	−1.5 ± 1.0	−1.5 ± 1.0	−1.4 ± 1.0
Osteocalcin (ng/mL)	18.8 ± 12.2	20.5 ± 12.1	18.2 ± 12.3	20.1 ± 11.6	18.2 ± 6.8	20.3 ± 13.5
BAP (*μ*g/mL)	20.2 ± 7.4∗∗	23.1 ± 8.4	19.3 ± 6.6∗∗	23.0 ± 7.1	19.7 ± 6.5∗	22.2 ± 9.0
CTX (pmol/L)	4426.2 ± 3546.9∗	5439.5 ± 3143.0	4105.3 ± 2162.7∗	5324 ± 3395	4002.9 ± 2484.6∗	4909.3 ± 3112.0
PTH (pg/mL)	22.2 ± 15.6∗∗	35.5 ± 18.5	20.0 ± 15.8∗∗	33.3 ± 16.4	16.6 ± 13.0∗∗	31.5 ± 19.0
25OHD (ng/mL)	30.2 ± 8.8∗∗	14.4 ± 3.6	33.1 ± 8.6∗∗	16.8 ± 7.8	37.2 ± 8.4∗∗	18.8 ± 5.9

Values are mean ± SD. BMI = body mass index; BAP = bone alkaline phosphatase; CTX = C-telopeptides; PTH = parathormone; 25OHD = 25-hydroxyvitamin D. ∗∗*P* < 0.01 versus subjects with 25OHD values lower than their respective cutoff value. ∗*P* < 0.05 versus subjects with 25OHD values lower than their respective cutoff value.
